# Porous Iron Oxide Core–Gold Satellite Nanocomposite: A Cost‐Effective and Recyclable Solution for Photocatalytic Wastewater Treatment

**DOI:** 10.1002/smsc.202300266

**Published:** 2023-12-24

**Authors:** Thomas Myeongseok Koo, Hong En Fu, Jun Hwan Moon, Eunsoo Oh, Yeonbeom Kim, Min Jun Ko, Young Keun Kim

**Affiliations:** ^1^ Department of Materials Science and Engineering Korea University Seoul 02841 Republic of Korea; ^2^ Department of Radiology Northwestern University Chicago IL 60611 USA

**Keywords:** advanced oxidation processes, Au, Fe_3_O_4_, organic pollutants, photo‐Fenton reactions, porous core‐satellite nanocomposites, selective etching

## Abstract

Organic pollutants in wastewater pose serious threats to human health and the environment. Nevertheless, potent strategies are being devised to tackle this critical issue. Specifically, advanced oxidation processes driven by solar energy and photocatalysts show promise for the degradation of organic pollutants. In particular, porous nanomaterials are effective as photocatalysts because of their high surface area, light‐harvesting properties, and ability to generate reactive oxygen species. To further expedite the development of feasible and sustainable organic‐pollutant‐degrading schemes, this article reports a strategy rooted in selective core etching for fabricating a core‐satellite‐type porous Fe_3_O_4_–Au nanocomposite. The Fe_3_O_4_ core behaves as a semiconductor to generate electron–hole pairs, whereas the Au satellite units serve as hot‐electron donors under visible‐light irradiation. The nanocomposite exhibits exceptional photocatalytic activity, degrading methylene blue molecules by 97% in just 1 h under visible light, and maintains its photocatalytic performance even after five rounds of recycling. Moreover, the nanocomposite achieves 90% tetracycline decomposition within 2 h. In addition to exhibiting noteworthy photocatalytic activity, the nanocomposite is magnetically separable, facilitating its recovery and reuse and exhibiting remarkable stability and cost‐effectiveness. These attributes underscore the practical viability of the core‐satellite‐type Fe_3_O_4_–Au nanocomposite in wastewater treatment.

## Graphical abstract

This article reports a porous Fe_3_O_4_ core–Au satellite nanocomposite prepared using a selective etching strategy for realizing advanced oxidation processes under visible light. The porous structure of the synthesized nanomaterial considerably enhances its photocatalytic activity, surface area, and light absorption. Notably, the nanocomposite exhibits remarkable stability while degrading organic pollutants, achieving high performance even in small quantities.

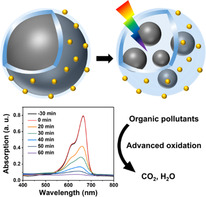

## 1 Introduction

Over the past few decades, efforts to combine dissimilar nanomaterials have unveiled new avenues for creating high‐performance materials with enhanced properties.^[^ ^1^, ^2^, ^3^ ^]^ Notably, innovative structures such as yolk–shell, rattle‐type, and porous or hollow core‐shell configurations have been devised to achieve beneficial features such as improved resistance to deformation, large surface area, electromagnetic wave absorption capability, and the propensity to control the storage and release of specific molecules within their internal spaces.^[^ ^4^, ^5^, ^6^, ^7^ ^]^ These versatile structures have been applied in secondary batteries, drug delivery systems, and catalysis. However, the high‐temperature annealing treatment of these structures—which is vital for enabling phase transitions and nucleation—hinders the production of regularly sized units and uniform dispersibility, thereby limiting their expected merits.^[^ ^8^, ^9^, ^10^ ^]^

Various porous structures exhibit enhanced light reflection and scattering in their interiors, thereby inducing light absorption and reducing the transport distance of the generated charges, facilitating charge separation.^[^ ^11^, ^12^, ^13^, ^14^, ^15^ ^]^ These characteristics make them particularly advantageous for photocatalytic applications. Photocatalysts play a vital role in sustainable energy development and environmental protection by enabling hydrogen and oxygen evolution reactions through sunlight or generating reactive oxygen species (ROS). Notably, transition‐metal photocatalysts are adept at promoting advanced oxidation processes (AOPs) and potent chemical treatment procedures designed to remove organic molecules from wastewater with photo‐Fenton reactions. The reactions are a process that generates ROS using light, hydrogen peroxide (H_2_O_2_), and Fe ions. Metal‐oxide semiconductors and novel metal nanomaterials such as titania, magnetite, zinc oxide, gold, and silver have often been employed as raw materials for facilitating photo‐Fenton reactions.^[^ ^16^, ^17^, ^18^, ^19^ ^]^ These materials have been combined to achieve a broad light absorption range or cooperatively boost ROS formation reactions to enhance efficiency.

**Scheme 1:**
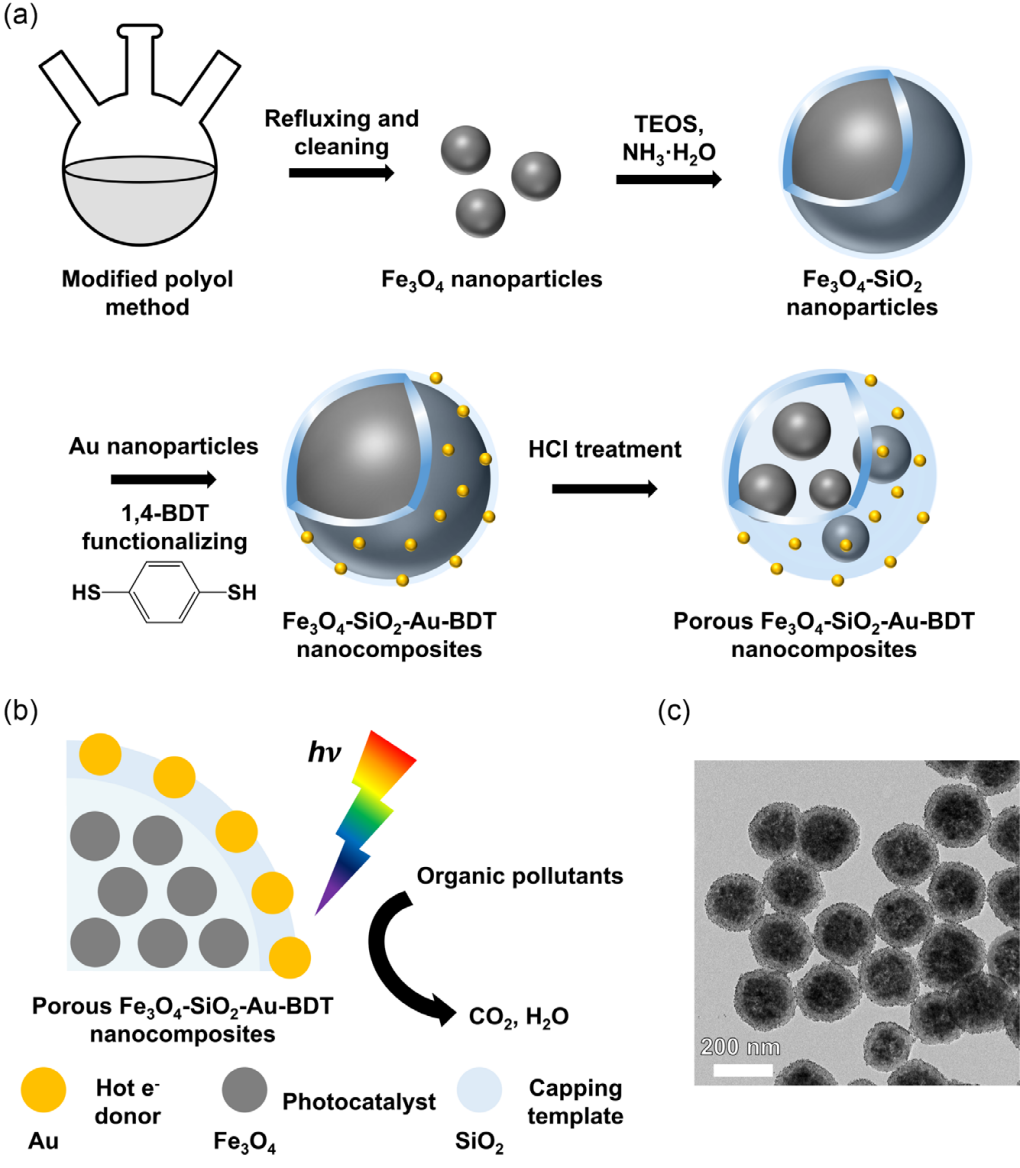
Synthesis and operation of a porous Fe_3_O_4_–SiO_2_–Au–BDT (PFAB) nanocomposite designed for AOPs. Schematics illustrating the a) synthesis of the PFAB catalyst and b) the role of each catalyst component in generating ROS under visible‐light irradiation to degrade organic pollutants in water to CO_2_ and H_2_O. c) TEM image of the PFAB nanocomposites.

In this study, we developed the porous core–satellite nanocomposite using a selective etching strategy to implement an AOP by leveraging its photocatalytic behavior (see **Scheme** **1** for relevant schematics and a transmission electron microscopy (TEM) image). The designed nanocomposite, which comprised Au satellite units around a porous magnetite (Fe_3_O_4_) core, exhibited uniform morphology, excellent stability, and a wide surface area. The Fe_3_O_4_ semiconductor core generated electron–hole pairs under visible‐light irradiation, producing ROS with the Fenton reaction. The porous structure could rapidly separate the generated charges, providing a clear advantage over nonporous nanomaterials. Furthermore, the magnetic properties of the nanocomposite rendered it reusable.

The silica shell acts as a solid template, creating internal spaces as Fe_3_O_4_ undergoes etching. These spaces promote reflection and scattering of light within, increasing the chances of light absorption by Fe_3_O_4_. Additionally, the silica shell protects Fe_3_O_4_ against any physical or chemical damage. Hot electrons, which were generated by the Au satellite units under visible‐light illumination through interband and intraband transitions, directly contributed to ROS production or were transferred to the Fe_3_O_4_ core, facilitating the Fenton reaction. Benzene‐1,4‐dithiol (1,4‐BDT) molecules stabilized the Au satellite units and boosted light absorption because of their aromatic ring. Overall, the synergistic interactions between the subunits of the porous Fe_3_O_4_–SiO_2_–Au–BDT nanocomposite (denoted as PFAB herein) were expected to promote the photo‐Fenton reaction of an AOP.

## 2 Results and Discussion

### 2.1 Synthesis of Porous Fe_3_O_4_ Core‐Au Satellite Nanocomposites

We initiated the process by synthesizing 170 nm‐sized Fe_3_O_4_ nanoparticles via the modified polyol method to fabricate porous‐based photocatalysts.^[^ ^20^ ^]^ The nanoparticles exhibited a monodisperse distribution but showed poor dispersibility in an aqueous solution owing to self‐aggregation (Figure S1, Supporting Information). Notably, the hydrodynamic size of the Fe_3_O_4_ nanoparticles, as determined by dynamic light scattering (DLS), was significantly larger in water than in ethanol. Therefore, the nanoparticles were encased in a 22.5 nm‐thick silica shell using the Stöber method to mitigate this issue. The silica shell enhanced the nanoparticle stability in aqueous solutions and acted as a protective barrier, thereby preventing the agglomeration of the core and shielding it from chemical damage. We attempted to etch the Fe_3_O_4_ core in a 1 м HCl solution at 45 °C; however, this proved challenging, primarily due to the passivation effect of the silica shell (Figure S2a, Supporting Information). TEM imaging revealed that under acidic conditions for 1 h, only a portion of the cores was completely etched (pink dashed circle in Figure S2a, Supporting Information), while the rest remained intact. This outcome was undesirable because it compromised the magnetic and photocatalytic properties of the nanocomposite.

Therefore, we decorated the Au nanoparticles with an average diameter of 3.5 nm onto the silica surface to create the porous core,^[^ ^21^ ^]^ yielding the Fe_3_O_4_–SiO_2_–Au nanocomposite (denoted as FA). As expected, the hydrodynamic size of the nanoparticles gradually increased as they were coated with silica and then decorated with Au satellite units (Figure S1e, Supporting Information). As mentioned above, the Au satellite units were then utilized to expedite the core etching and overcome the challenge. Au nanoparticles were expected to function as catalysts in redox reactions, thereby facilitating the decomposition of Fe_3_O_4_ into iron chloride in the presence of HCl. However, a complication arose when the Au satellite units detached and aggregated on the silica surface during etching (blue dashed circle in Figure S2b, Supporting Information) because HCl could also react with the nanosized Au units.^[^ ^22^ ^]^ The Au satellite units initially reacted with HCl to form gold chloride ions, which penetrated the silica interior and abstracted electrons from Fe_3_O_4_ before being reduced back to Au. In this process, Au preferentially grew by attaching to existing satellite units rather than by self‐nucleation. Therefore, the Au satellite units were functionalized with 1,4‐BDT to address this issue and stabilize them under the harsh conditions employed for selectively etching Fe_3_O_4_, thereby yielding the nanocomposite denoted as FA‐BDT (FAB) (Figure S2c, Supporting Information). We suggested that the lone electron pair of the thiol group in 1,4‐BDT provided electrons to the Au ions, facilitating their reduction and the electron extraction from Fe_3_O_4_. The silica shell acted as a barrier and prevented direct electron exchange between Fe_3_O_4_ and Au. Additionally, the thiolated aromatic ring molecules exhibited an antenna effect, which helped enhance the absorption properties of adjacent materials and bolster photocatalytic activity.^[^ ^23^ ^]^ This functionalization strategy permitted the selective etching of the Fe_3_O_4_ core units, resulting in porous FAB (PFAB) nanocomposites with uniformly sized porous cores and retained Au satellite units (Figure S2d, Supporting Information).

**Figure 1:**
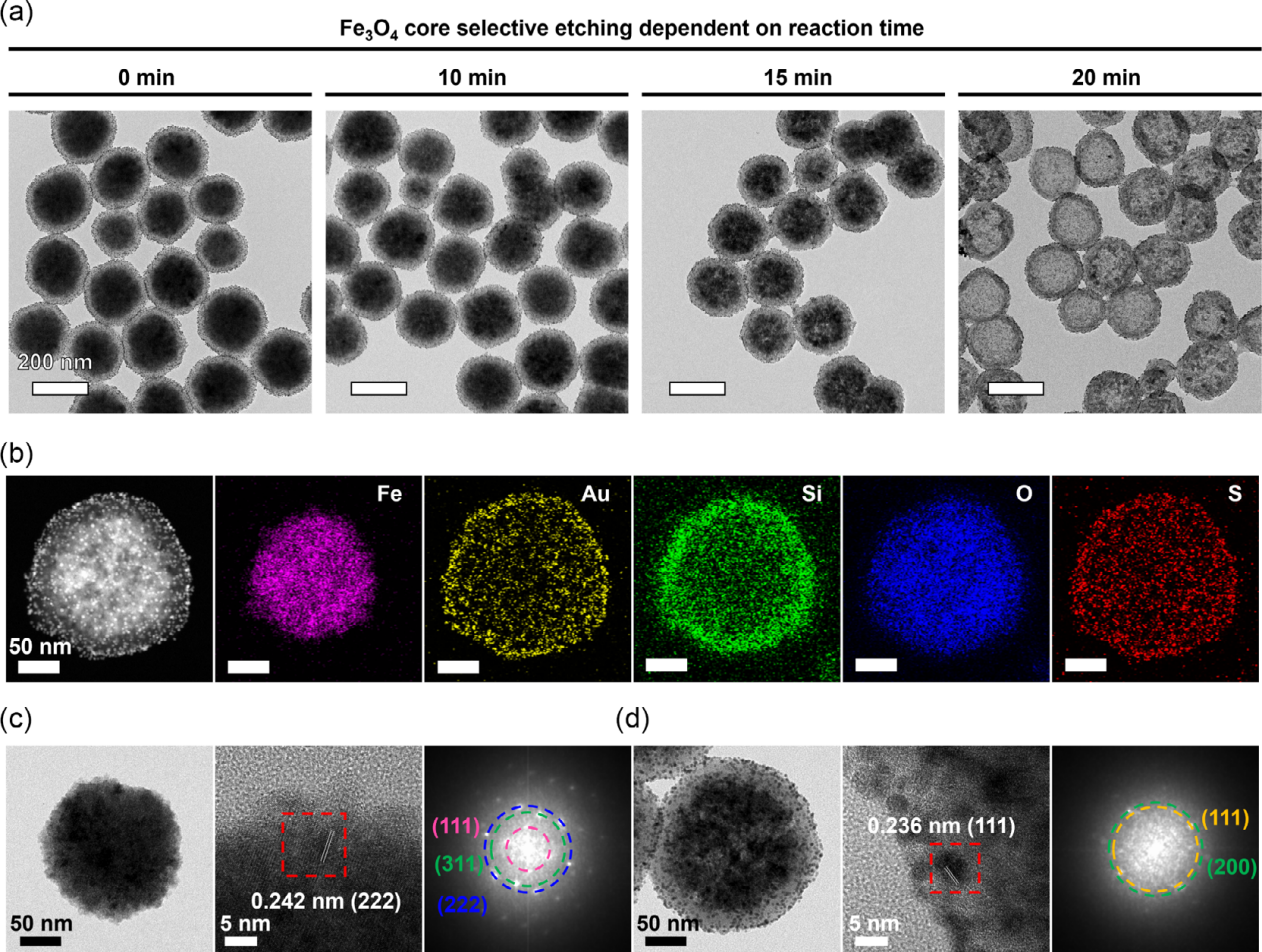
Morphology and microstructure of the PFAB nanocomposite. a) Reaction‐time‐dependent porosity of the Fe_3_O_4_ core units. b) HAADF‐STEM image and EDS‐based elemental maps of PFAB. c,d) HR‐TEM images and FFT patterns of the Fe_3_O_4_ core and Au satellite units. The FFT patterns correspond to the square area marked by red dashes in the HR‐TEM images.

We controlled the porosity of the Fe_3_O_4_ cores by adjusting the reaction time, as demonstrated in **Figure** **1**a. The reaction system appeared dormant for the first 10 min; however, internal pores and pseudo‐hollow cores emerged 15 and 20 min, respectively, after the reaction began. The sample etched for 20 min experienced significant depletion of Fe_3_O_4_, known for its magnetic and photocatalytic properties, leading us to anticipate poor performance. Therefore, we selected the 15‐min etched sample for further analysis to test the hypothesized ability of the porous structure to achieve enhanced photocatalytic performance. High‐angle annular dark‐field scanning TEM (HAADF‐STEM) imaging and energy‐dispersive spectroscopy (EDS)‐based elemental mapping of the PFAB nanocomposite (Figure 1b) validated the presence of the Fe_3_O_4_ core units, silica shell, and Au satellite units, in addition to the distribution of the sulfur component of the 1,4‐BDT molecules along with the Au units. We further examined the microstructures of the core and satellite units by analyzing the d‐spacing values of Fe_3_O_4_ and Au by high‐resolution TEM (HR‐TEM) and the plane groups within the fast Fourier transform (FFT) patterns (Figure 1c,d).

### 2.2 Characteristics of PFAB Nanocomposites

**Figure 2:**
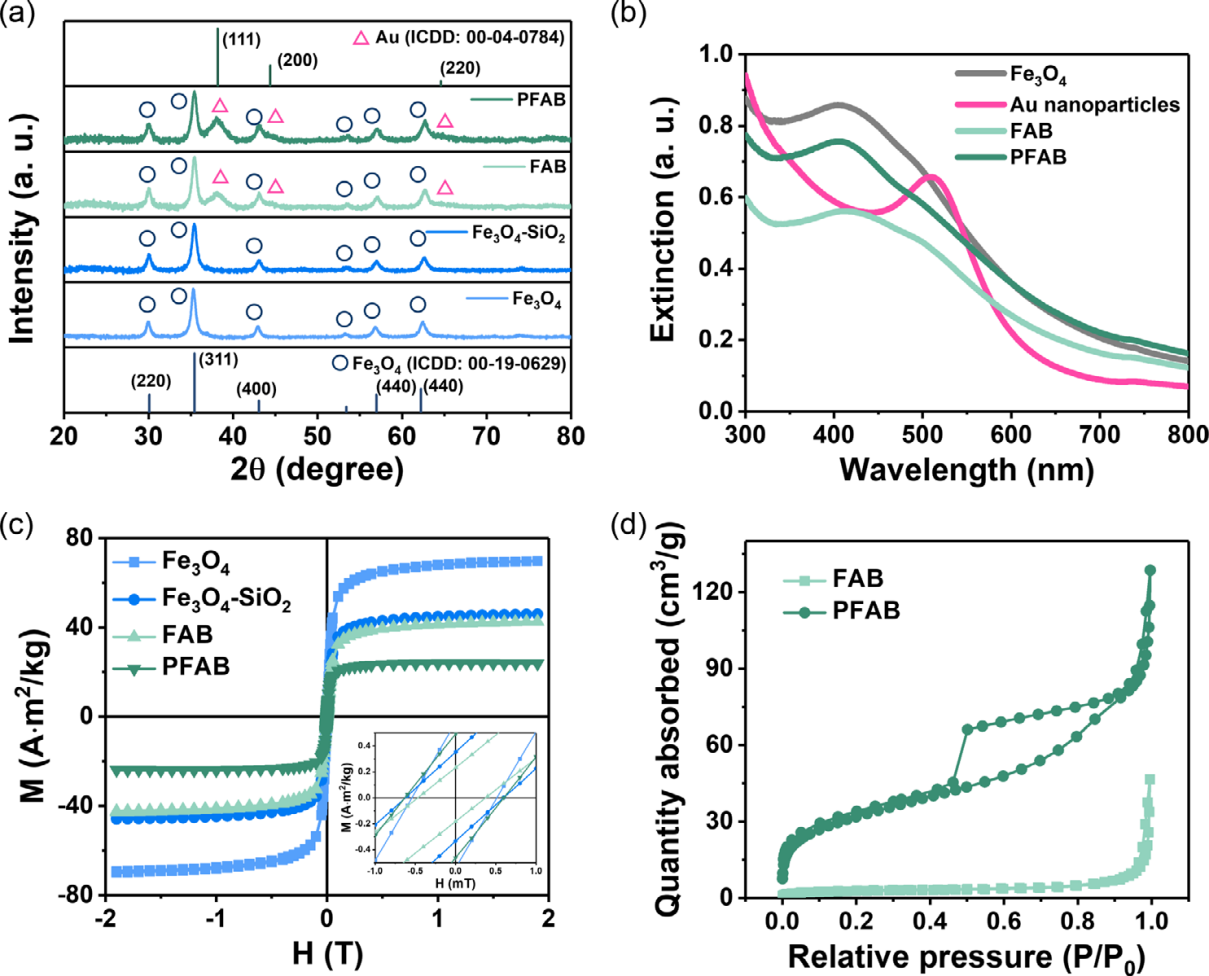
Characteristics of PFAB nanocomposites. a) X‐ray diffractometry patterns of the nanomaterials produced during the synthesis procedures, indexed to ICDD card nos. 00‐19‐0629 (Fe_3_O_4_) and 00‐04‐0784 (Au). b) UV–vis spectra of the nanomaterials. c) Magnetic hysteresis loops of the nanomaterials, with the inset showing an expanded view of the near‐zero field. d) N_2_ adsorption–desorption isotherms of FAB and PFAB.

The acquired X‐ray diffractometry (XRD) data, which indicated the crystal structures of the nanoparticles and nanocomposites, matched the expected patterns of Fe_3_O_4_ and Au provided in the ICDD database (**Figure** **2**a). We calculated the crystallite size of Fe_3_O_4_ in the Fe_3_O_4_ nanoparticle, Fe_3_O_4_–SiO_2_ nanoparticle, and FAB nanocomposite to be ≈12.3 nm using the Debye–Scherrer equation; however, that for PFAB decreased to 11.3 nm, primarily because the etchant preferred to react with the grain boundaries due to their higher surface energy. The iron oxide core exhibited mesocrystalline properties, and its bonding surface underwent etching after ≈20 min, yielding particles with a rattle‐type configuration. The crystallite size of Au in FAB and PFAB was calculated to be 3.9 and 3.6 nm, respectively, aligning with those estimated by TEM. The difference suggested that the satellite units were single crystalline and could not be fully protected by the functionalizing molecules during etching.

Investigation of the light‐absorption properties of the nanocomposites revealed composition‐dependent behavior (Figure 2b). Peaks appeared at 400 nm owing to the bandgap of the Fe_3_O_4_ core units. Pristine Au nanoparticles exhibited a peak at 520 nm owing to surface plasmon resonance, with absorption below this peak corresponding to interband 5*d* → 6*sp* excitation.^[^ ^24^ ^]^ However, the peaks of FAB and PFAB were almost indistinguishable, primarily because of the pronounced intensity of Fe_3_O_4_. Subsequently, FAB and PFAB with identical concentrations were subjected to UV–vis spectroscopic analysis to ascertain the ability of the porous core to enhance light scattering. PFAB exhibited higher absorption than FAB, attributed to the light reflection and scattering from the internal pores within the silica shell. In the case of FAB, incident light was absorbed once, whereas PFAB had more chances to absorb even scattered or reflected light, resulting in a higher absorbance.

Figure 2c presents the magnetic characteristics of those nanomaterials. Fe_3_O_4_ nanoparticles exhibited a magnetization of 69.7 A m^2^ kg^−1^, which gradually decreased with the successive reactions to 45.9, 42.6, and 23.8 A m^2^ kg^−1^. The dominant magnetic attributes of the ferrimagnetic core units were diluted upon adding silica and gold, which attenuated the magnetization per unit mass. Additionally, the loss of Fe_3_O_4_ during etching further mitigated the magnetization, particularly that of PFAB. The nanomaterials exhibited a magnetic coercivity of ≈0.5 mT and displayed superparamagnetic‐like behavior, which helped them achieve high dispersibility after removing the external magnetic field. This property of PFAB permitted accessible collection following its use in AOPs.

We also obtained the nitrogen (N_2_) adsorption–desorption isotherms for the nanocomposites to confirm the presence of internal pores in PFAB, as shown in Figure 2d. The results indicated that PFAB and FAB exhibited typical mesoporous and nonporous characteristics, respectively. We calculated the specific Brunauer–Emmett–Teller (BET) specific surface area and Barrett–Joyner–Halenda (BJH) pore volume (Figure S3 and Table S1, Supporting Information). PFAB exhibited an approximately 11.7 times higher surface area (115.6 m^2^ g^−1^) than FAB, along with a 2.65‐fold higher pore volume (0.1814 cm^3^ g^−1^) during the Fe_3_O_4_ core etching. Consequently, PFAB offered more active ROS generation sites than FAB ones.^[^ ^25^ ^]^

### 2.3 Surface State Analysis During the Chemical Reaction

**Figure 3:**
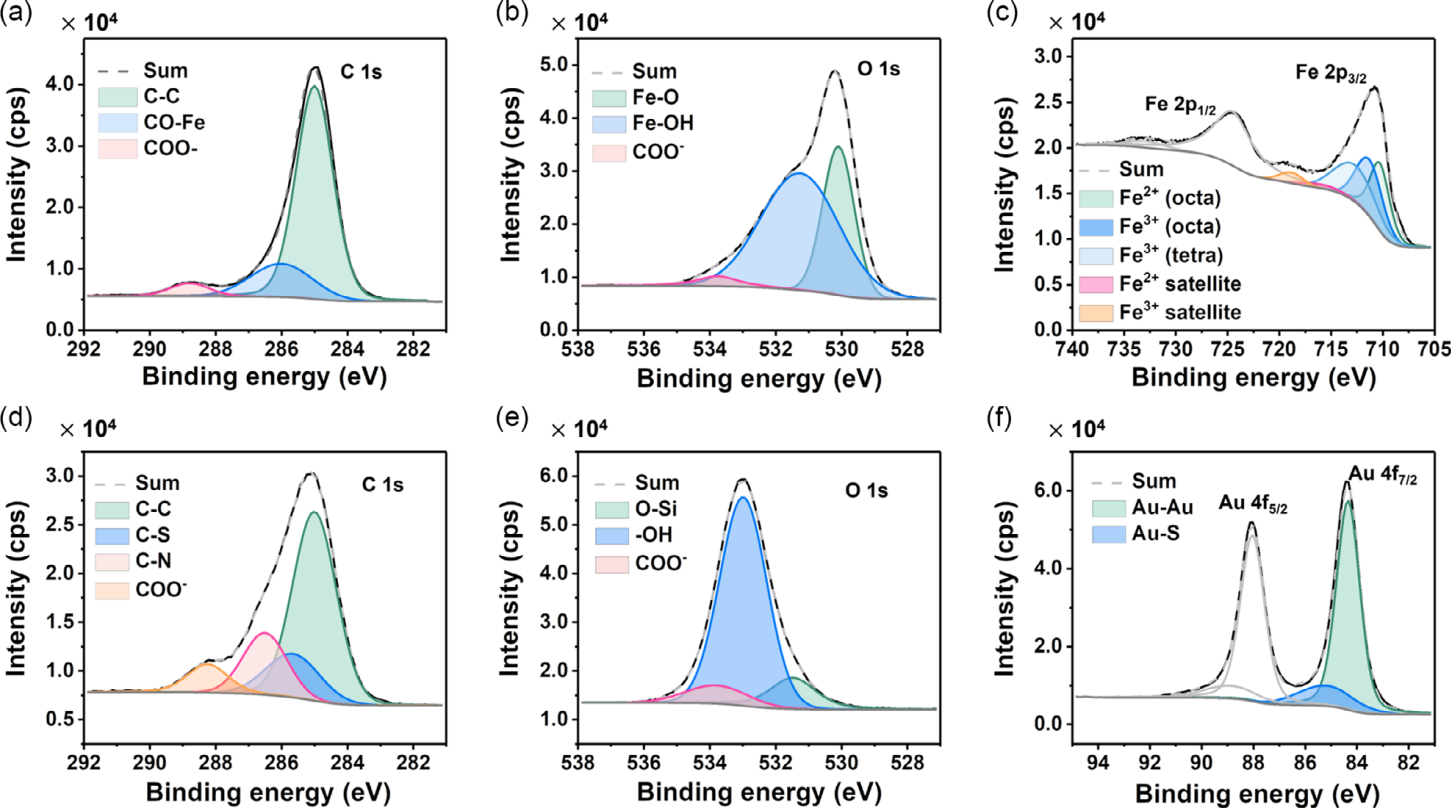
X‐ray photoelectron spectroscopy profiles of the Fe_3_O_4_ cores and the PFAB nanocomposites. a,d) C 1*s* spectra of the Fe_3_O_4_ cores and PFAB nanocomposites, respectively. b,e) O 1*s* spectra of the Fe_3_O_4_ cores and the PFAB nanocomposites, respectively. c) Fe 2*p* spectrum of the Fe_3_O_4_ cores. f) Au 4*f* spectrum of the PFAB nanocomposites.

X‐ray photoelectron spectroscopy comprehensively explored the surface states of Fe_3_O_4_ and PFAB (XPS; **Figure** **3**). We calibrated all XPS profiles against the C—C bonding peak at 285 eV. The C 1*s* spectra showed peaks corresponding to C—C bonding, CO—Fe bonding between the acetate and surface of Fe_3_O_4_, and COO^−^ groups at 285, 286, and 288.7 eV, respectively (Figure 3a).^[^ ^20^ ^]^ The O 1*s* spectra showed a peak associated with Fe—O bonding at 530.1 eV, representing the lattice oxygen of the Fe_3_O_4_ crystallite structure, and those corresponding to Fe—OH bonding on the surface and COO^−^ groups at 531.3 and 533.7 eV, respectively (Figure 3b). Fe atoms generated three prominent peaks stemming from the Fe_3_O_4_ crystallite structure (Figure 3c). The core nanoparticles had an inverse spinel structure, as verified by XRD, with the oxygen atoms arranged in a face‐centered cubic lattice. Within this structure, Fe atoms existed in tetrahedral and octahedral sites coordinated by oxygen, with Fe^2+^ ions occupying the octahedral sites (710.4 eV) and Fe^3+^ ions occupying both the octahedral (711.6 eV) and tetrahedral sites (713.3 eV) in equal proportions.^[^ ^26^ ^]^ The approximate ratio of Fe^2+^ to Fe^3+^ ions in this structure (0.5) is consistent with the chemical formula of Fe_3_O_4_. Notably, the C1s spectrum of PFAB nanocomposites lacked the peak associated with CO–Fe binding, as the photoelectrons could not entirely escape the SiO_2_ shell (Figure 3d). The Fe 2*p* spectrum could not be captured for the same reason. Instead, C–S and C–N peaks appeared at 285.6 and 286.5 eV, originating from 1,4‐BDT and poly(vinylpyrrolidone) (PVP).^[^ ^27^, ^28^ ^]^ The presence of COO^−^ groups from trisodium citrate, which acted as stabilizers for the Au satellite units, was also confirmed. Furthermore, the peak shifted toward lower binding energies than Fe_3_O_4_, owing to the higher electronegativity of Au than that of Fe. The O 1*s* spectrum revealed bindings related to the silica shell rather than Fe_3_O_4_ (Figure 3e). In particular, the peaks at 531.5 and 533.0 eV corresponded to the –OH groups on the silica surface and the O–Si binding in silica, respectively,^[^ ^29^ ^]^ and the citrate‐derived COO^−^ groups continued to exhibit the peak at 533.7 eV. The Au 4*f* spectrum showed peaks related to Au–Au and Au–S binding at 84.3 and 85.2 eV, respectively, confirming the bonding between 1,4‐BDT and Au (Figure 3f).^[^ ^30^ ^]^

Additionally, we conducted Fourier transform infrared (FT‐IR) spectroscopy to analyze the chemical bonding of the nanomaterials (Figure S4, Supporting Information). Peaks associated with Fe—O bonding (578 cm^−1^), COO^−^ bidentate bonding with Fe (1397 and 1622 cm^−1^), C–O–C groups (1090 cm^−1^), and –CH_3_ groups (2850 and 2930 cm^−1^) were exhibited by all samples.^[^ ^21^, ^31^ ^]^ Moreover, peaks related to Si–O (798 and 1188 cm^−1^) and Si–OH (991 cm^−1^) from silica were visible, except for the as‐synthesized Fe_3_O_4_ nanoparticles.^[^ ^32^, ^33^ ^]^ Notably, the peak representing the surface hydroxyl groups on the metal oxide (1388 cm^−1^) was faint for Fe_3_O_4_–SiO_2_ but more prominent for the other samples. The bare Fe_3_O_4_ nanoparticles, rich in surface hydroxyl groups, were expected to facilitate the generation of hydroxyl radicals^[^ ^34^ ^]^; however, the silica shells appeared to mask these groups. Consequently, the area of the exposed Fe_3_O_4_ increased during etching, potentially boosting the performance of PFAB. Overall, the identified molecular bonding modes correspond to the chemical reactions during the PFAB synthesis.

### 2.4 Photocatalytic Activity

**Figure 4:**
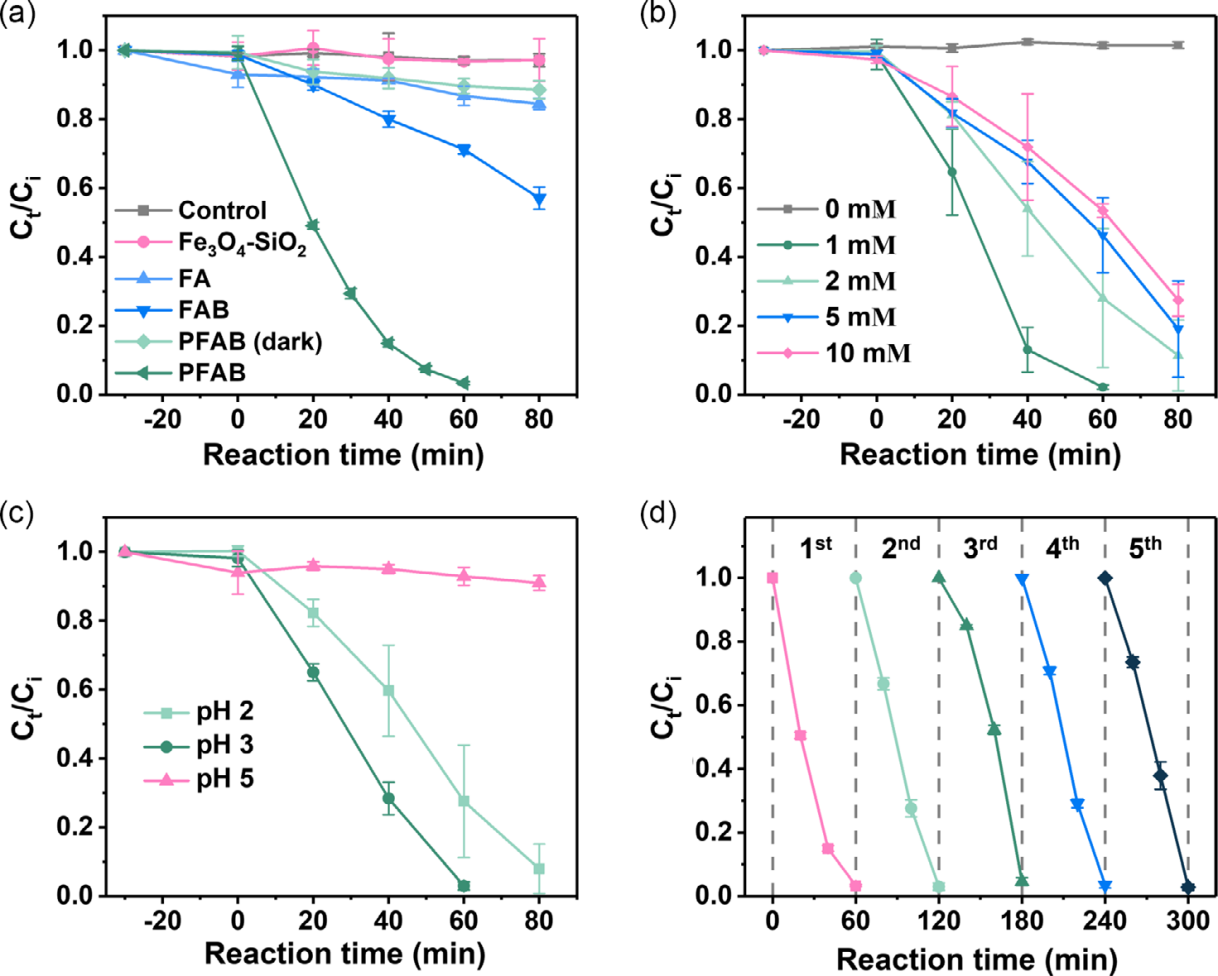
Photo‐Fenton degradation of MB using the PFAB nanocomposites. a) MB removal efficiencies of the different nanomaterials under light/dark conditions (MB = 10 mg L^−1^, catalyst dose = 0.05 g L^−1^, H_2_O_2_ concentration = 1 mм, initial pH = 3.0, *n* = 3). b,c) Dependence of the MB degradation efficiency of PFAB on (b) H_2_O_2_ concentration and (c) pH. The other conditions were identical to those in (a). d) Reusability experiments of PFAB nanocomposites for MB degradation.

The components of PFAB—porous Fe_3_O_4_ core units, a silica template, and Au satellite units—were postulated to exhibit synergistic photocatalytic effects for degrading organic pollutants. Methylene blue (MB), an organic dye, was selected as a model organic pollutant for the degradation experiments. MB is conducive to naked‐eye observations, and UV–vis spectroscopy can capture its intense absorption. As MB is a photoactive dye, we assessed its light‐induced decomposition before conducting the photo‐Fenton degradation. After light irradiated the MB solution, approximately ≈1.78% decomposition was observed within 80 min (Figure S5, Supporting Information). The degradation of MB over time was studied using the different nanocomposites as photocatalysts (**Figure** **4**a); we excluded the as‐synthesized Fe_3_O_4_ nanoparticles due to their poor aqueous dispersibility. The Fe_3_O_4_–SiO_2_ core‐shell nanoparticles exhibited a remarkably low MB removal efficiency (≈3%) because although they were capable of participating in photo‐Fenton degradation, the surface of Fe_3_O_4_ offered a small area for generating ROS, given the presence of the silica shell. This value was similar to that of the catalyst‐free control group, mainly because the strong absorption of Fe_3_O_4_ interfered with the H_2_O_2_ dissociation by the absorbed light, thereby inhibiting the generation of ·OH radicals. In addition, to validate the photocatalytic efficacy of Au satellite units, we conducted an MB decomposition experiment using pseudo‐hollow core nanocomposites and light, excluding H_2_O_2_ (Figure S6, Supporting Information). The results confirmed an efficiency of ≈5.39% and indicated a more dominant role of the Fenton reaction involving Fe_3_O_4_ compared to the photocatalytic reaction of Au satellites.

FA demonstrated a slightly higher MB removal efficiency (≈15.5%) than Fe_3_O_4_–SiO_2_ over 80 min. The Au satellite units contained electron transition pathways that permitted interband and intraband transitions, which enabled them to generate electron–hole pairs. These pairs either induced energy transfer to Fe_3_O_4_ for the Fenton reaction or helped degrade H_2_O_2_, thereby generating ·OH radicals. FAB exhibited a higher MB removal efficiency of 43.0% over 80 min because 1,4‐BDT—the FAB functionalization agent—featured an aromatic ring capable of absorbing photon energy from the light source and transferring it to the grafted Au.^[^ ^35^ ^]^

PFAB nanocomposites achieved an impressive MB degradation efficiency of ≈97% within 60 min (Figure S7, Supporting Information); this value is comparable to that of several recently reported nanocomposite photocatalysts that can efficiently degrade organic pollutants with a low catalyst loading (**Table** **1**). The porous structure significantly reduced the distance over which electron–hole pairs traveled within the catalyst to reach the surface, resulting in enhanced removal efficiency. Additionally, the internal pores amplified the light reflection and scattering, thereby increasing the likelihood of light absorption and further boosting the removal efficiency. Notably, this cumulative effect vanished under dark conditions, and only the Fenton reaction occurred, resulting in an efficiency of ≈11.5%. We calculated the reaction rate constant *k* for all reaction systems as following Equation (1) (Figure S8, Supporting Information)

(1) $$\ln\left(\frac{C_{i}}{C_{t}}\right)=kt$$

where *C*_*i*_ is the initial concentration of MB, as measured by UV–vis spectroscopy, and *C*_t_ is the concentration at a specific time. The *k* value of PFAB (4.10 × 10^−2^ min^−1^) was 8.9 times higher than that of FAB (4.60 × 10^−3^ min^−1^), which had the second highest *k* value among the investigated systems.

**Table 1:** The Photocatalytic efficiency of PFAB compared with those of previously reported pollutant‐degrading nanocomposite catalysts

Catalysts	Catalyst loading [mg mL^−1^]	Pollutants	Initial amount of pollutants [mg L^−1^]	Time [min]	Light source	Removal efficiency [%]	References
PFAB nanocomposites	0.05	MBa)	10	60	300 W, Xe lamp	97	This study
Fe_3_O_4_–ZnFe_2_O_4_–ZnO	0.1	MB	10	60	300 W, Xe lamp	97	[26]
MNPb)@SiO_2_@TiO_2_	0.56	MB	3.2	30	80 W, Hg lamp	95	[44]
Ag/SrMoO_4_	0.5	MB	5	35	300 W, Xe lamp	84	[45]
Fe_3_O_4_/g‐C_3_N_4_	0.33	MB	5	100	500 W, Xe lamp	90	[46]
3NCQDc)s/TiO_2_	0.1	MB	10	60	300 W, Xe lamp	93	[47]
ZIFd)‐8/MnFe_2_O_4_	0.4	TCe)	20	80	300 W, Xe lamp	99	[48]
Fe‐g‐C_3_N_4_/Bi_2_WO_6_	0.4	TC	10	120	500 W, Xe lamp	97	[37]
g‐C_3_N_4_/Fe_3_O_4_/Ag_3_PO_4_	1	MGf)	10	12	sunlight	100	[49]
rGOg)–ZnO	0.8	4‐BPh)	10	180	30 W, UV lamp	99	[50]

a) methylene blue.

b) magnetic nanoparticle.

c) N‐doped carbon quantum dot.

d) zeolite imidazole framework.

e) tetracycline.

f) malachite green.

g) reduced graphene oxide.

h) 4‐bromophenol.

We subsequently adjusted the H_2_O_2_ concentration and pH to optimize the MB‐degrading capacity of PFAB. When the H_2_O_2_ concentration was varied (Figure 4b), no reaction occurred without H_2_O_2_, but the reaction rate decreased as the H_2_O_2_ concentration exceeded 1 mм. These findings indicated that the nanocomposite could not generate ROS without H_2_O_2_ and exhibited a ROS scavenging effect in the presence of excess H_2_O_2_, which reduced the ·OH radical concentration.^[^ ^36^ ^]^ However, the nanocomposites are cost‐effective because small amounts of H_2_O_2_ can activate them.^[^ ^37^ ^]^ The pH plays a critical role in the generation of ROS. For instance, in the classical Fenton reaction conducted at low pH values, H^+^ ions quench ·OH radicals and form H_3_O_2_^+^ ions, which have low reactivity with Fe ions. In contrast, Fe ions form undesirable ferric sludge at high pH values, and ·OH radicals exhibit low oxidation potential.^[^ ^38^ ^]^ The pH of the photo‐Fenton system investigated in the present study was determined to be 3 for optimal MB removal (Figure 4c). Additionally, PFAB nanocomposites maintained their photocatalytic performance after five rounds of recycling (Figure 4d), with no significant morphological differences observed after that (Figure S9, Supporting Information). Furthermore, PFAB nanocomposites exhibited magnetic properties that facilitated loss‐free recovery.

### 2.5 Scavenging Behavior and Identification of ROS Radicals in MB Degradation

We conducted scavenger experiments to ascertain the predominant ROS radicals in the investigated photo‐Fenton system. We selected isopropanol (IPA), triethanolamine (TEOA), silver nitrate (AgNO_3_), and p‐benzoquinone (p‐BQ) to scavenge hydroxyl radicals (·OH), holes (h^+^), electrons (e^−^), and superoxide ions ($O_{2}^{-}$), respectively. Each scavenger was added to the MB solution, and the resulting degradation was compared with a scavenger‐free blank sample.

**Figure 5:**
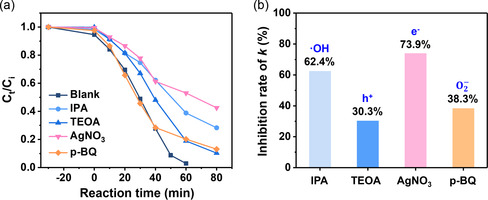
Scavenger tests were conducted with respect to photo‐Fenton degradation of MB. a) Effects of several ROS scavengers on PFAB‐catalyzed MB degradation. b) Inhibition rate of *k* by different species (Inhibition rate of *k* (%) = 1−*k*/*k*_blank_).

As shown in **Figure** **5**a, the results of the scavenger experiments indicated that the p‐BQ‐ and TEOA‐containing samples degraded MB by ≈90% over 80 min. However, the IPA‐ and AgNO_3_‐integrated specimens exhibited lower degradation. These results were subsequently used to calculate the extent to which the reaction rate was inhibited compared with that in the blank model (Figure 5b). AgNO_3_, which decreased the efficiency from 97% to 57.5%, achieved the highest inhibition effect among the scavengers, with *k* being reduced by 73.9%. IPA exhibited an inhibition rate of 62.4%, followed by p‐BQ (38.3%) and TEOA (30.3%). These results indicated that electrons were primarily responsible for generating ROS in the photo‐Fenton system. The reactions related to the ROS generation in this photo‐Fenton system can be expressed as follows:

(2) $${\text{Fe}}_{3}O_{4}+\text{hν}\to e^{-}+h^{+}$$

(3) $$H_{2}O_{2}+e^{-}+H^{+}\to \cdot \text{OH}+H_{2}O$$

(4) $$H_{2}O_{2}+{\text{Fe}}^{2+}+e^{-}+H^{+}\to \cdot \text{OH}+H_{2}O+{\text{Fe}}^{3+}$$

(5) $$2H_{2}O_{2}+\text{hν}\to O_{2}+2H_{2}O$$

(6) $$O_{2}+e^{-}\to O_{2}^{-}$$

(7) $$O_{2}^{-}+e^{-}+2H^{+}\to H_{2}O_{2}$$

$O_{2}^{-}$ and ·OH were the two main ROS responsible for the decomposition of MB into CO_2_ and H_2_O. Consequently, impairment of the ROS generation directly led to a reduction in removal efficiency. For the same reason, scavenging ·OH radicals impeded the MB degradation. However, p‐BQ and TEOA had a lesser impact than the other scavengers, suggesting that •OH was the dominant oxidant involved in MB degradation within the system. Hence, the pivotal role of electrons lies in triggering the Fenton reaction rather than in the generating $O_{2}^{-}$. The holes generated by Fe_3_O_4_ primarily reacted with Fe^3+^ and H_2_O_2_ as follows, yielding $O_{2}^{-}\text{:}$

(8) $${\text{Fe}}^{2+}+h^{+}\to {\text{Fe}}^{3+}$$

(9) $$H_{2}O_{2}+h^{+}\to O_{2}^{-}+2H^{+}$$

**Scheme 2:**
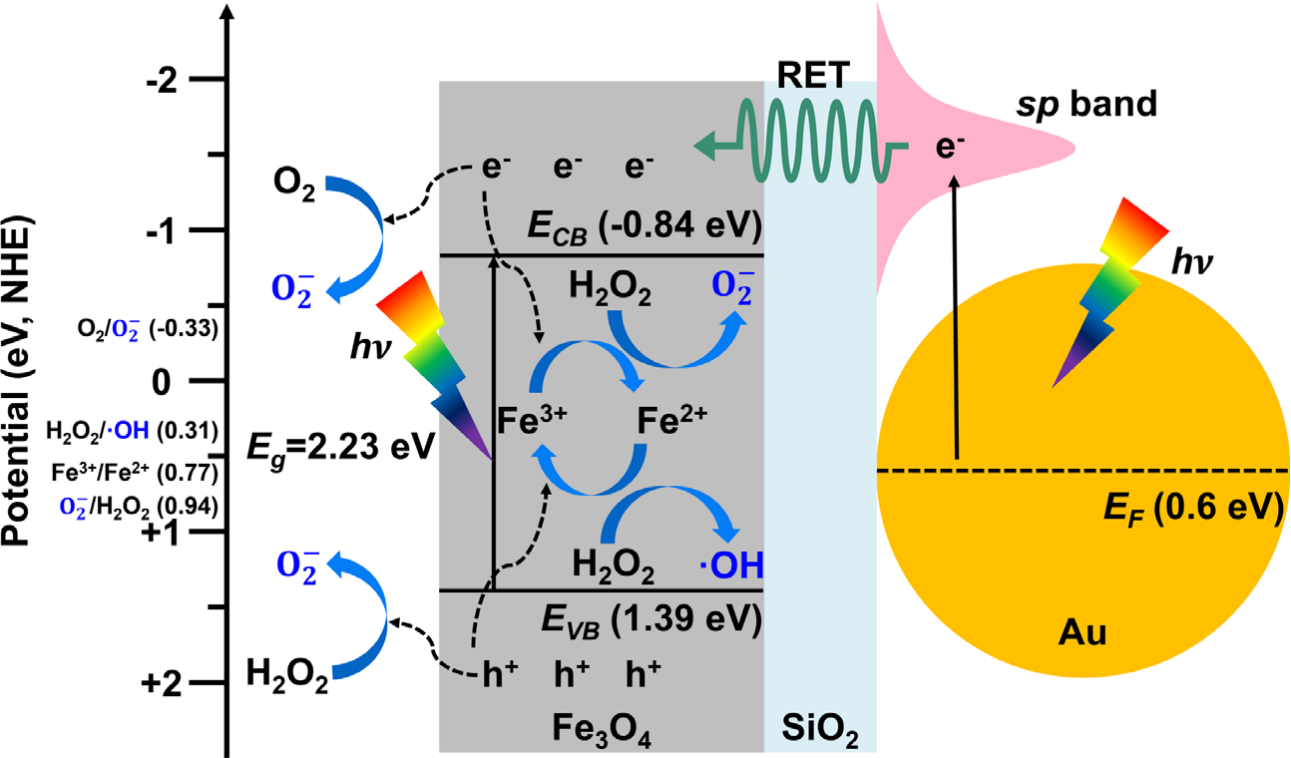
Proposed photocatalysis mechanism of the PFAB‐based photo‐Fenton system for generating ROS.

Here, as both TEOA‐ and p‐BQ‐containing samples exhibited similar trends, it suggests that the major role of holes was generating $O_{2}^{-}$, rather than actively participating in the Fenton‐like reaction. We collated these findings and proposed a photocatalysis mechanism for generating ROS in the PFAB‐based photo‐Fenton system (**Scheme** **2**). The bandgap of the Fe_3_O_4_ core units (2.23 eV) was estimated by UV–vis absorption spectrophotometry and Tauc plot analysis (Figure S10, Supporting Information). Subsequently, we determined the position of the valence band of Fe_3_O_4_ to be 1.39 eV by XPS (Figure S11, Supporting Information). The Fermi level of the Au satellite units was assumed to be 0.6 eV, as reported previously.^[^ ^39^ ^]^ When the PFAB nanocomposite was irradiated with light, the Fe_3_O_4_ core and Au satellite units generated electron–hole pairs, respectively. The excited electrons in the *sp* band of the Au satellite reacted with the O_2_ dissolved in H_2_O or transferred the energy to the Fe_3_O_4_ core units. Considering the photocatalytic performance of Au satellites (Figure S6, Supporting Information), it appears unlikely that Au directly generated superoxide ions. However, as depicted in Figure 4a, performance enhancement was observed when Fe_3_O_4_ cores were included, indicating that the energy absorbed by Au was transferred to Fe_3_O_4_. Despite the insulating properties of the SiO_2_ layer hindering direct electron transfer (DET), the phenomenon of energy transfer through resonance energy transfer (RET) is known.^[^ ^40^ ^]^ RET is more effective when the donor and acceptor are separated rather than attached, and SiO_2_ serves as a spacer for causing RET in this context. This energy transfer phenomenon promoted the Fenton reaction within Fe_3_O_4_, thereby helping generate ROS more effectively than from Au. Additionally, Au absorbed light and excited electrons at a longer wavelength than Fe_3_O_4_ (Figure 2b). Consequently, PFAB nanocomposites could absorb a broader range of light than single nanomaterials, which increased their removal efficiency. Electrons and holes were more likely to escape to the surface because of the porous structure, reducing the likelihood of recombination within Fe_3_O_4_. As a result, the generated electrons and holes reacted with O_2_, Fe ions (Fe^2+^ and Fe^3+^), and H_2_O_2_ to produce superoxide and hydroxyl radicals. SiO_2_ contained pores, allowing ions and ROS to move freely.^[^ ^41^, ^42^ ^]^ Through this, ROS penetrated the SiO_2_ and oxidized MB attached to the surface of PFAB, breaking it down into CO_2_ and H_2_O.

Tetracycline, widely used as an antibiotic in farms to protect livestock from diseases, is often discharged into wastewater, causing environmental pollution. Therefore, the ability of PFAB to degrade tetracycline was also evaluated (Figure S12, Supporting Information). The PFAB‐based photo‐Fenton system achieved more than 90% decomposition of tetracycline in 2 h, demonstrating its potential for practical applications.

## 3 Conclusion

A core‐satellite nanocomposite (PFAB) of porous Fe_3_O_4_ and Au was synthesized using selective etching to perform AOP‐style organic pollutant decomposition. Essentially, 170 nm‐diameter Fe_3_O_4_ nanoparticle core units were coated with a 22.5 nm‐thick silica shell, which was subsequently decorated with Au nanoparticles having an average diameter of 3.5 nm as satellite units; subsequently, we functionalized the Au satellites with 1,4‐BDT. HCl was used to selectively etch the core and make it porous, which led to a significant increase in the surface area and light absorption, leading to a remarkable enhancement in the MB removal efficiency. The Au satellite units played a crucial role in improving the photocatalytic activity via energy transfer and ROS generation. The silica shell stabilized the core and preserved magnetic properties, ensuring its reusability. Additionally, its insulating properties induced RET rather than DET, reducing energy loss during energy transfer and increasing removal efficiency. The combination of silica and gold, which have adequate dispersibility in aqueous solutions, enhanced the overall stability of the nanocomposite. Owing to these characteristics, PFAB demonstrated performance comparable with that of several recently reported nanocomposite catalysts, even in minuscule quantities, and maintained its abilities and morphology during five reuse cycles. Specifically, the nanocomposites exhibited outstanding photocatalytic activity, degrading MB by 97% in just 1 h and tetracycline by 90% in 2 h under visible light. This report highlights the feasibility of using PFAB to degrade tetracycline, an actual antibiotic model. It presents a novel method for creating porous materials and an environmentally friendly wastewater treatment approach.

## 4 Experimental Section

4.1

4.1.1

##### Materials

Iron chloride hexahydrate (FeCl_3_•6H_2_O), sodium acetate (CH_3_COONa), PVP (*M*_w_ = ≈55 000), an ammonium hydroxide solution (NH_4_OH, 28%), tetraethyl orthosilicate (TEOS; Si(OC_2_H_5_)_4_), (3‐aminopropyl)triethoxysilane (APTES; H_2_N(CH_2_)_3_Si(OC_2_H_5_)_3_), gold chloride trihydrate (HAuCl_4_•3H_2_O), trisodium citrate dihydrate (HOC(COONa)(CH_2_COONa)_2_•2H_2_O), sodium borohydride (NaBH_4_), 1,4‐BDT (C_6_H_6_S_2_), H_2_O_2_ (30%), H_2_SO_4_, HCl (37%), and MB were obtained from Sigma‐Aldrich. Ethylene glycol ((HOCH_2_CH_2_)_2_O) was purchased from Alfa Aesar. Distilled water was prepared using a Millipore Direct‐Q UV 3 device.

##### Preparation of Nanocomposite Photocatalyst

Fe_3_O_4_ nanoparticles were synthesized using a modified polyol method.^[^ ^20^ ^]^ To that end, iron chloride hexahydrate (2 mmol) was dissolved in distilled water (150 mmol), and sodium acetate (6 mmol) was dissolved in ethylene glycol (50 mL). The two solutions were mixed under vigorous magnetic stirring, and the resulting mixture was heated to 200 °C. After refluxing for 3.5 h, the products were purified five times with ethanol and then dispersed in ethanol. Core–satellite‐structured Fe_3_O_4_–SiO_2_–Au nanoparticles were synthesized using the Stöber method and silane chemistry.^[^ ^21^ ^]^ To that end, the iron oxide nanoparticles (30 mg) and PVP (470 mg) were dissolved in a solution containing ethanol (60 mL), ammonium hydroxide solution (3 mL), and distilled water (9 mL). TEOS (60 μL) was subsequently added to the mixture, and the solution was agitated for 2 h. The ensuing Fe_3_O_4_–SiO_2_ core‐shell nanoparticles were washed five times with ethanol and redispersed in ethanol (30 mL). The core‐shell nanoparticle solution (10 mL) was then redispersed in isopropanol (10 mL). Subsequently, APTES (0.1 mL) was injected into the solution, and the mixture was sonicated at 80 °C for 4 h. The resulting amine‐functionalized Fe_3_O_4_–SiO_2_ nanoparticle solution was washed three times with ethanol and distilled water and then redispersed in distilled water (5 mL). Au nanoparticles were synthesized using a burst nucleation method.^[^ ^43^ ^]^ To that end, a solution of gold chloride trihydrate (2.5 mL, 5 mм) and trisodium citrate dihydrate (2.5 mL, 5 mм) was prepared in distilled water (45 mL). Subsequently, ice‐cold NaBH_4_ (1.5 mL, 0.1 м) was rapidly injected into the mixture, and the solution was agitated for 6 h. The functionalized Fe_3_O_4_–SiO_2_ nanoparticle solution (5 mL) was then injected into the Au solution (25 mL) and agitated overnight. The Fe_3_O_4_–Au core‐satellite‐type nanoparticle solution was washed using a permanent magnet and redispersed in a PVP solution (20 mL, 1 wt%) for stabilization. After being agitated for 2 h, the stabilized core‐satellite nanoparticles were cleaned three times and then redispersed in distilled water (FA, 20 mL). Subsequently, 1,4‐BDT (2 mL, 1 mм) was injected into the core–satellite nanoparticle solution for functionalization. The mixture was agitated for 3 h, washed thrice with distilled water, and redispersed in distilled water (FAB, 20 mL). Finally, the functionalized core–satellite nanoparticle solution (20 mL) was mixed with an HCl solution (20 mL, 2 м) to etch the Fe_3_O_4_ core units at 45 °C under weak ultrasonication. After the reaction, the solution was quenched in ice‐cold water, washed five times, and redispersed in distilled water (PFAB, 20 mL).

##### Characterization

The morphologies and microstructures of the nanomaterials were analyzed by HR‐TEM (JEM‐2100F; JEOL) and TEM‐EDS (X‐MAX^*n*^; HORIBA). The crystalline phases of the nanomaterials containing Fe_3_O_4_ and Au were assessed by XRD (Aeris Research; Malvern Panalytical). Powder XRD patterns were obtained using Cu Kα radiation (*λ* = 1.5406 Å) in the diffraction spectrum of 20° < 2*θ* < 80°. Surface analysis was performed using XPS (K‐Alpha; Thermo Fisher Scientific), FT‐IR spectroscopy (Nicolet iS50; Thermo Fisher Scientific), and DLS techniques (DLS Zetasizer; Nano ZS90; Malvern Panalytical). The magnetic properties were characterized by vibrating‐sample magnetometry (EV9‐380 V, Microsense) at room temperature. The absorption spectra of the nanomaterials and MB were acquired by UV–vis spectrophotometry (UV‐1800, Shimadzu) in the wavelength range 300–800 nm. The pore volume and specific surface area of the nanocomposites were evaluated using the BJH and BET method (Tristar II plus 3030; Micromeritics) with N_2_ gas.

##### Evaluation of Photocatalytic Activity

The photocatalytic activity of PFAB toward MB degradation was evaluated in the presence of an H_2_O_2_ and H_2_SO_4_ solution under visible light (300 W Xe arc lamp; DY‐Tech) at 25 °C. The preparation was performed under dark conditions. MB molecules were dispersed in an aqueous solution at a concentration of 0.01 mg mL^−1^, followed by adding the nanocomposite (1 mg) as a catalyst. Moreover, the pH of the solution was modified using sulfuric acid. The solution was then agitated for 30 min to achieve adsorption–desorption equilibrium. Subsequently, a specified amount of H_2_O_2_ was injected into the solution. The Xe lamp irradiated the solution without mixing at 25 °C. After each predetermined time interval, a 1 mL aliquot of the solution was collected and centrifuged for 1 min to obtain a pure MB solution, diluted with water (2 mL). UV–vis spectroscopy investigated the absorption peak at 664 nm and the total spectrum of the residual MB.

## Conflict of Interest

The authors declare no conflict of interest.

## Supporting information

Supplementary Material

## Data Availability

The data that support the findings of this study are available from the corresponding author upon reasonable request.
