# Supplementary material for: Porous Iron Oxide Core–Gold Satellite Nanocomposite: A Cost‐Effective and Recyclable Solution for Photocatalytic Wastewater Treatment
Source: Small Sci. 2023 Dec 24;4(2):2300266. doi: 10.1002/smsc.202300266 (PMC11935126; doi:10.1002/smsc.202300266)
Supplement: Supplementary file 1 — Supplementary Material [file SMSC-4-2300266-s001.pdf]

Supporting Information

Porous Iron Oxide Core-Gold Satellite Nanocomposite: A Cost-Effective and Recyclable Solution for Photocatalytic Wastewater Treatment

*Thomas Myeongseok Koo, Hong En Fu, Jun Hwan Moon, Eunsoo Oh, Yeonbeom Kim, Min Jun Ko, and Young Keun Kim**

T. M. Koo, H. E. Fu, J. H. Moon, E. Oh, Y. B. Kim, Y. K. Kim
Department of Materials Science and Engineering
Korea University
Seoul 02841, Republic of Korea
E-mail: ykim97@korea.ac.kr

M. J. Ko
Department of Radiology
Northwestern University
Chicago, IL 60611, USA

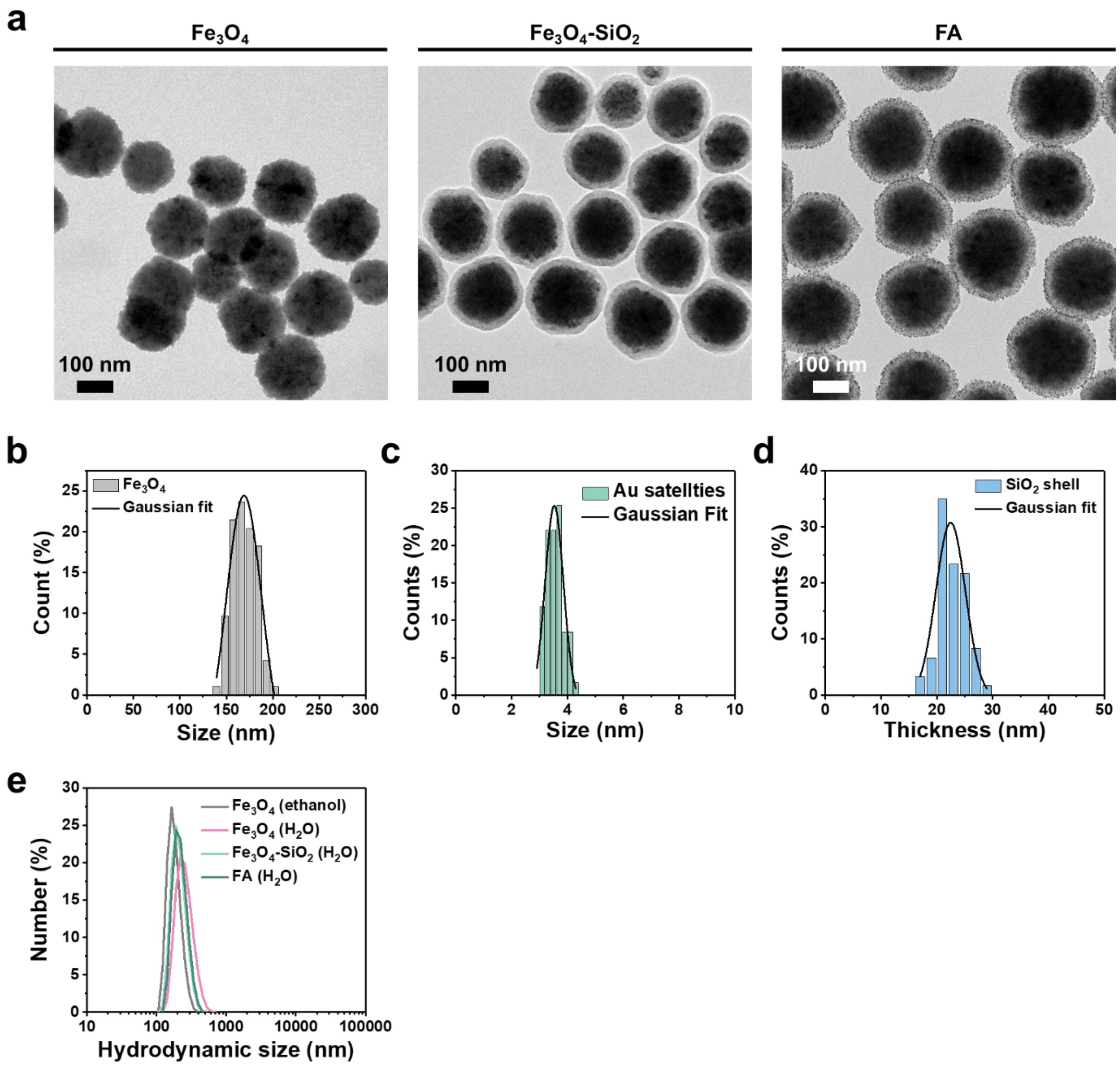

Figure S1. a) Transmission electron microscopy (TEM) images of Fe_3O_4, Fe_3O_4-SiO_2, and Fe_3O_4-SiO_2-Au (FA) nanomaterials. Size distribution of b) Fe_3O_4 nanoparticles, c) Au satellites, and d) thickness of SiO_2 shells from TEM images. e) Hydrodynamic size of each nanomaterial by measuring dynamic light scattering (DLS). The hydrodynamic size of Fe_3O_4 was measured in ethanol and H_2O, and the others were measured in H_2O.

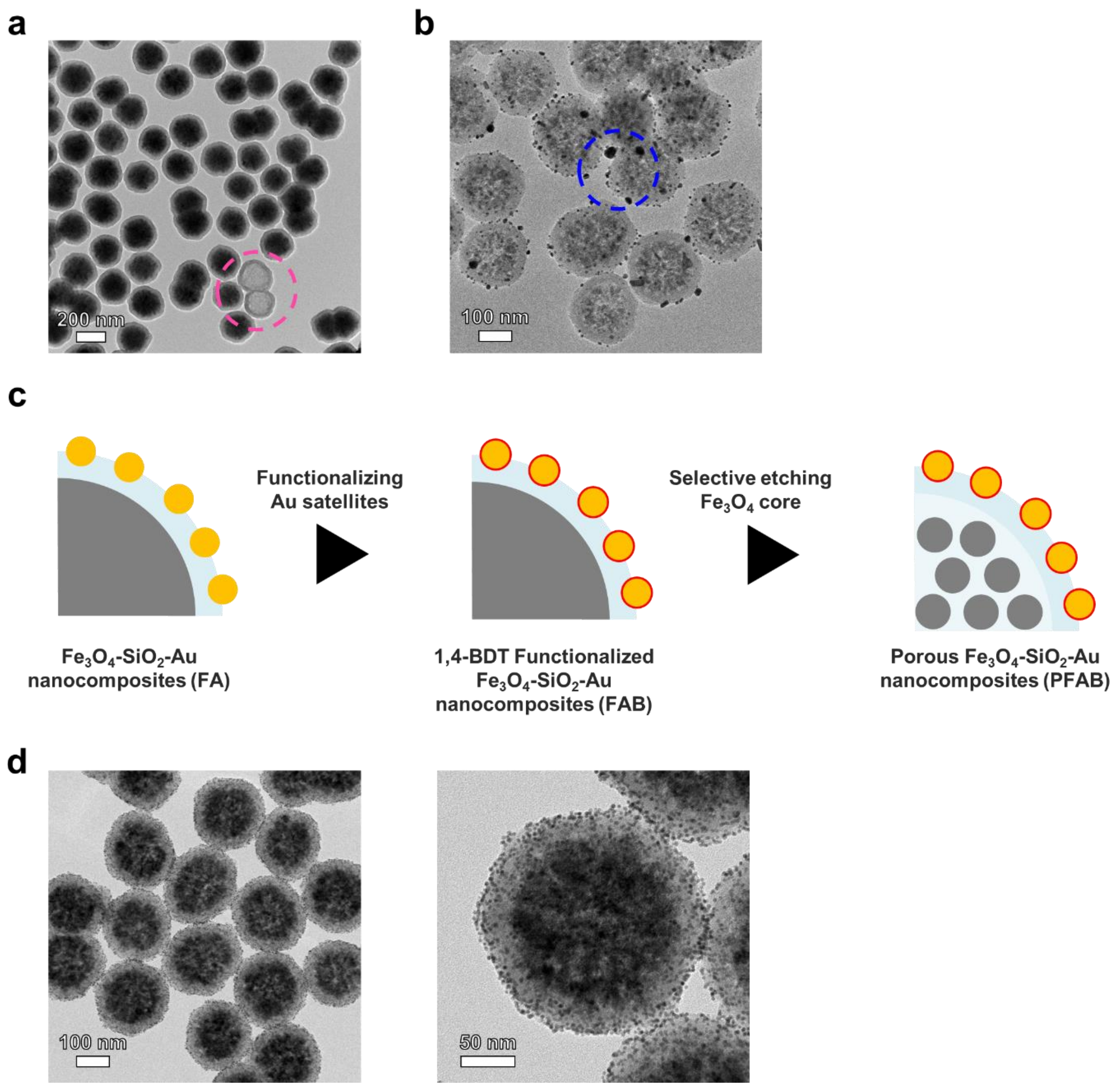

Figure S2. Au satellites and 1,4-BDT functionalization are required to produce uniform PFAB nanocomposites. a) A TEM image of Fe_3O_4-SiO_2 nanoparticles etched for an hour. b) A TEM image of Fe_3O_4-SiO_2-Au etched for 15 min without 1,4-BDT functionalization. c) Functionalizing the Au satellites procedures for protecting the satellites from chemical damage and agglomeration. d) TEM images of FAB etched for 15 min (PFAB nanocomposites).

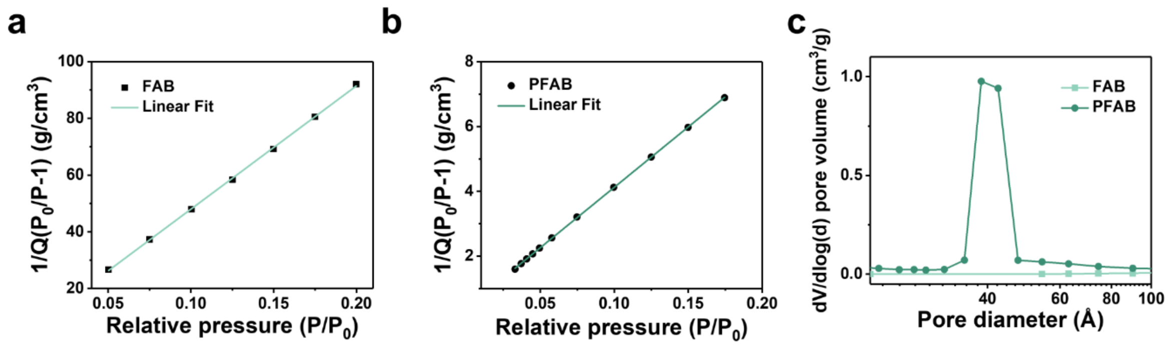

Figure S3. Brunauer-Emmett-Teller (BET) surface area plot of a) FA and b) PFAB nanocomposites. c) Barret-Jouner-Halenda (BJH) pore diameter distribution of FA and PFAB composites.

Table S1. Surface area and pore volume of FA and PFAB nanocomposites via measuring BET and BJH analyses.

Samples	Surface area [m^2 g^{-1}]	Pore volume [cm^3 g^{-1}]
FAB	9.906	0.06851
PFAB	115.6	0.1814

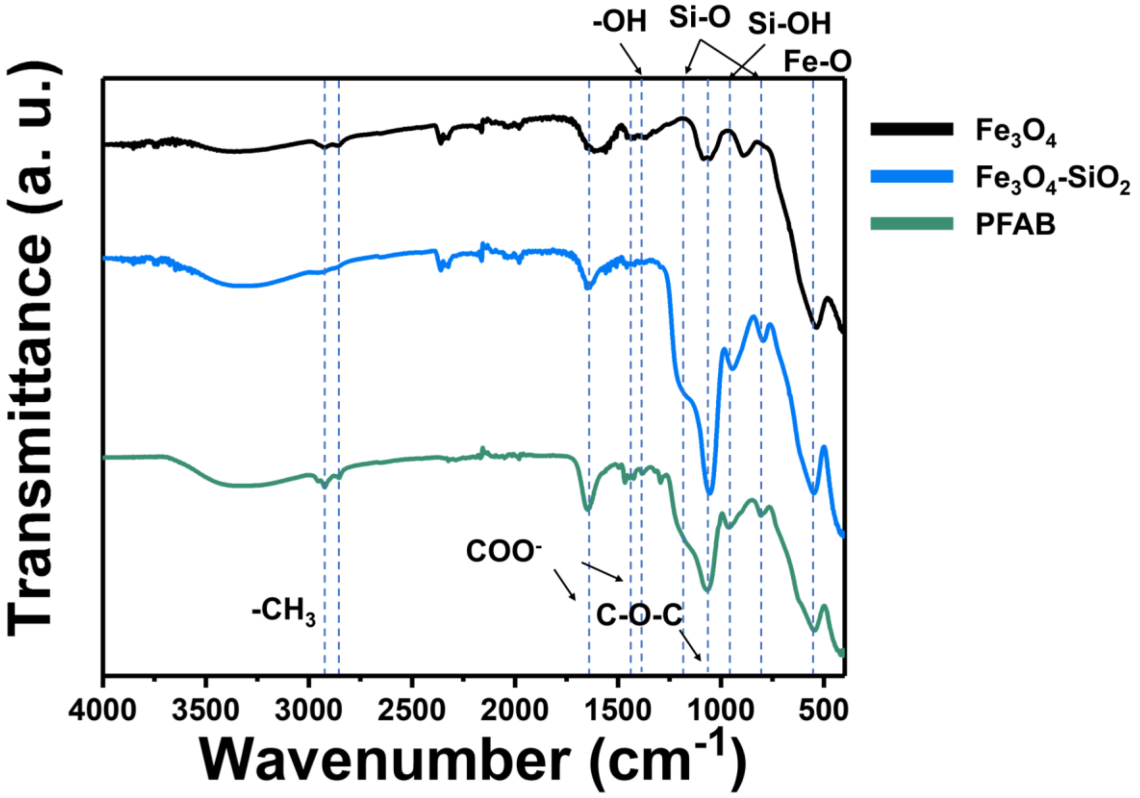

Figure S4. Fourier transformation infrared spectroscopy (FT-IR) spectra of Fe_3O_4, Fe_3O_4-SiO_2, and PFAB nanomaterials.

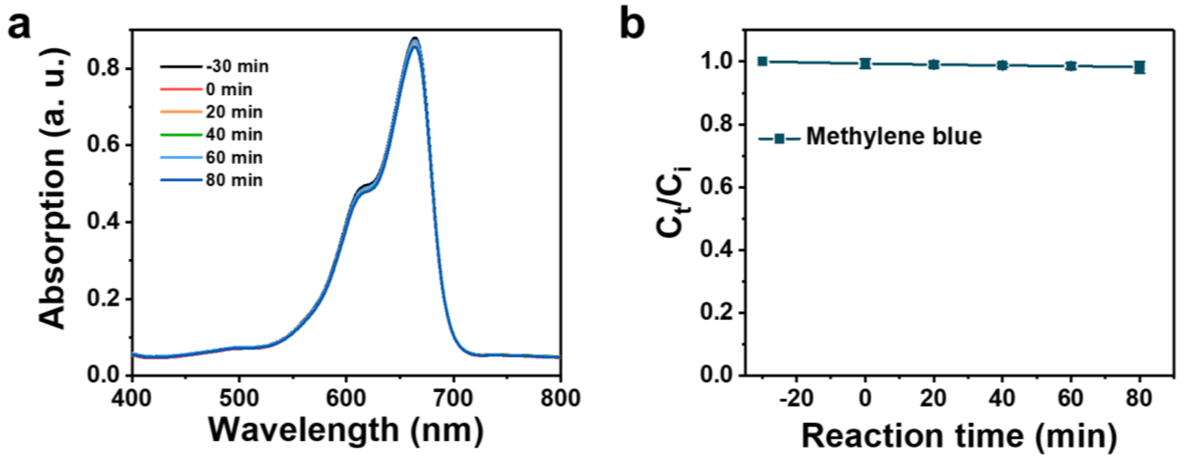

Figure S5. Photoactivity test of methylene blue (MB). a) UV-Vis absorption spectra of methylene blue (MB) according to light irradiation time. b) MB removal efficiency by light irradiation (MB = 10 mg L^{-1}, n = 3).

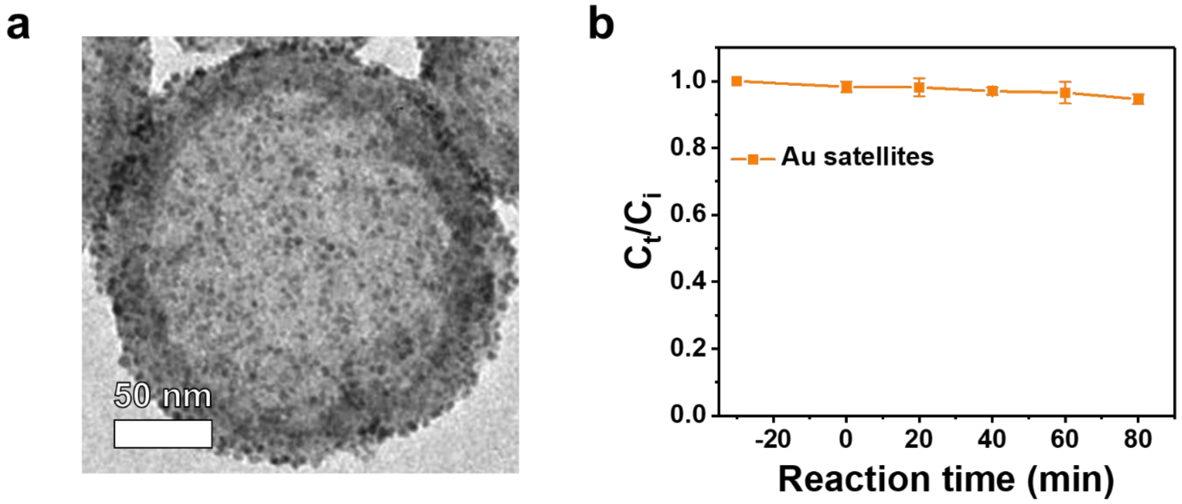

Figure S6. Photocatalytic activity on MB degradation of Au satellites without photo-Fenton reaction. a) A TEM image of pseudo-hollow core–Au satellite nanocomposites. b) MB removal efficiency of the photocatalytic effect by Au satellites. (MB = 10 mg L^{-1}, initial pH = 3.0, n = 3).

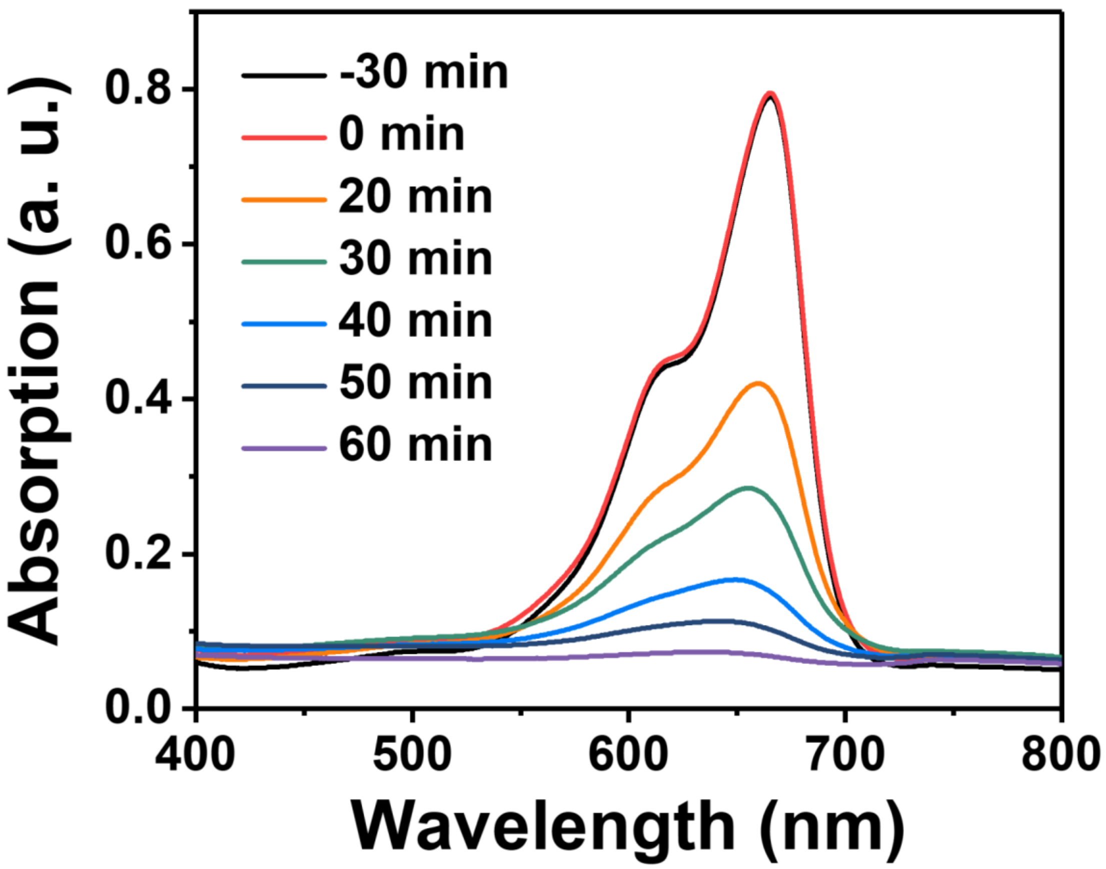

Figure S7. UV-Vis absorption spectra showed photo-Fenton degradation of MB by PFAB nanocomposites over time.

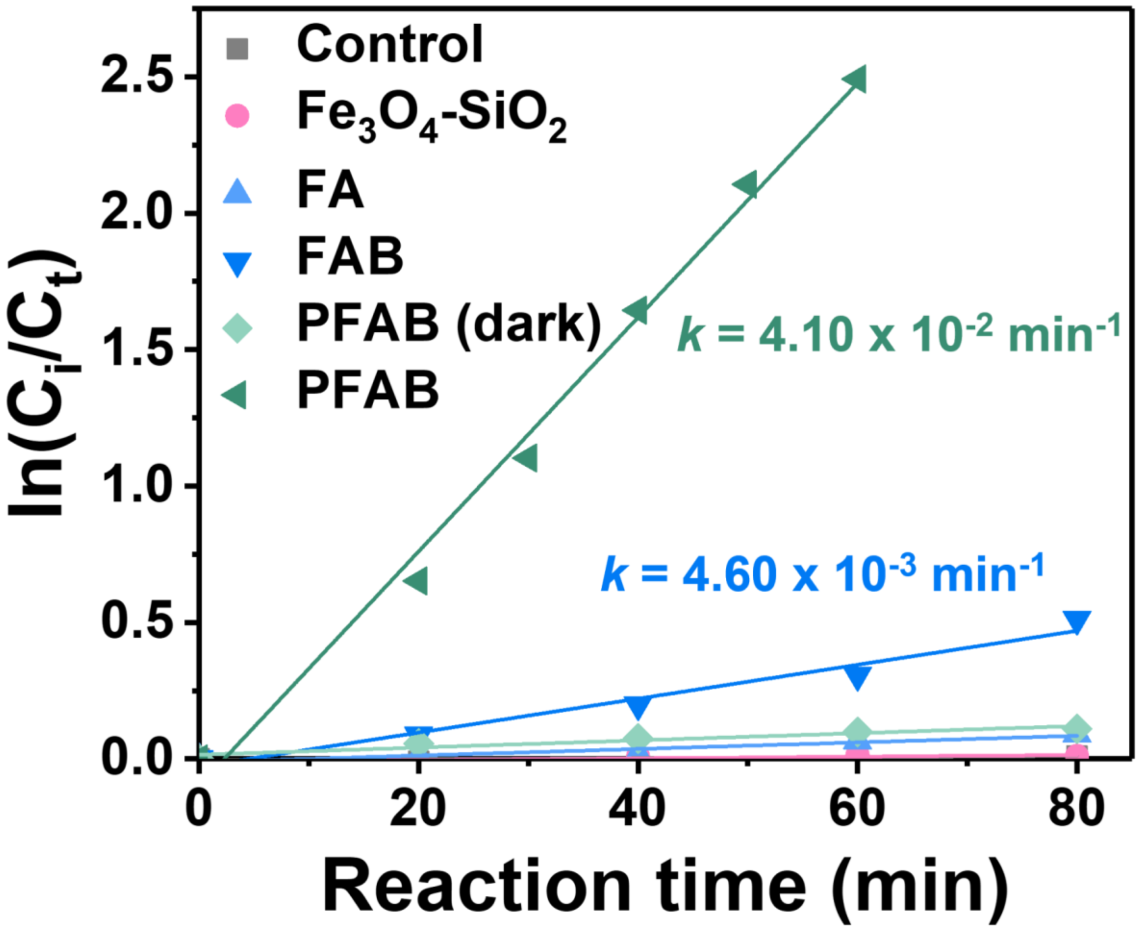

Figure S8. The reaction-rate constant (k) of MB degradation with the nanomaterials (k is the slope of $\ln(C_i/C_t)$-reaction time (min) graph).

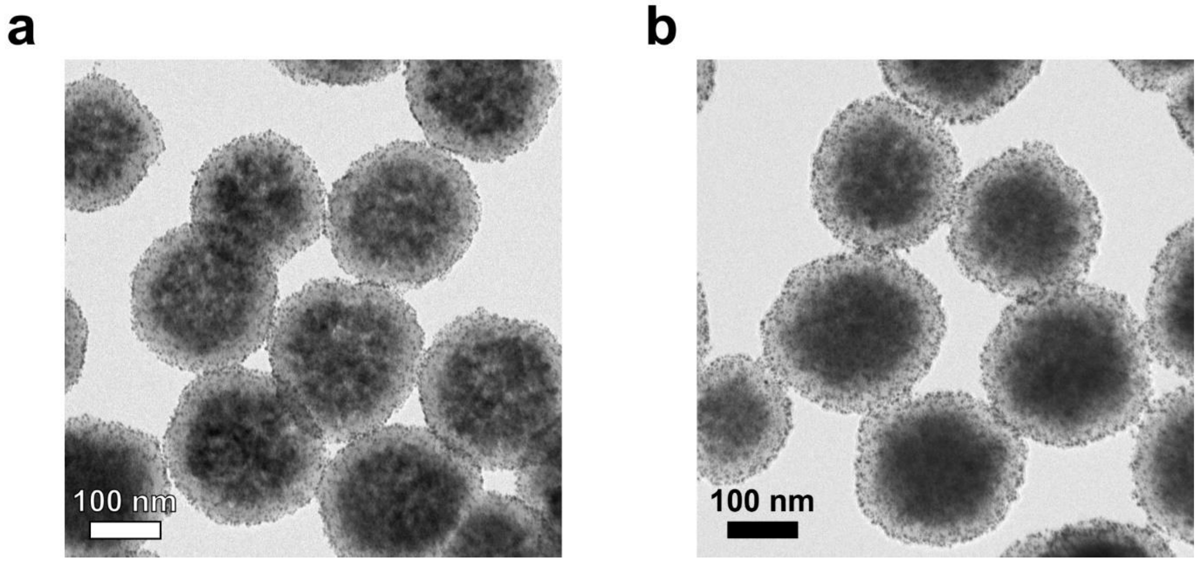

Figure S9. TEM images of PFAB nanocomposites: a) as-synthesized and b) after five consecutive use cycles.

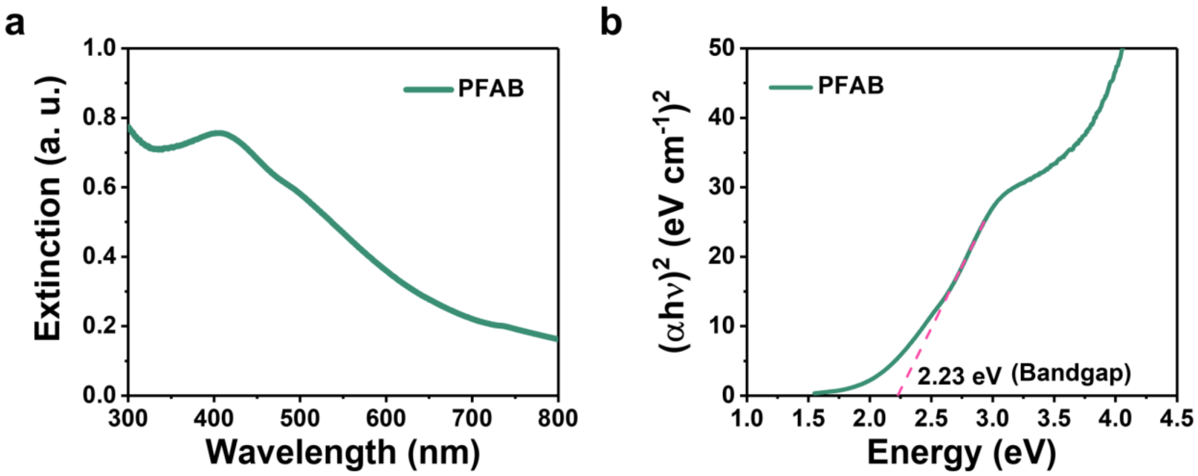

Figure S10. a) UV-Vis spectrum of PFAB catalysts. b) Bandgap energy of PFAB nanocomposites obtained by Tauc plot.

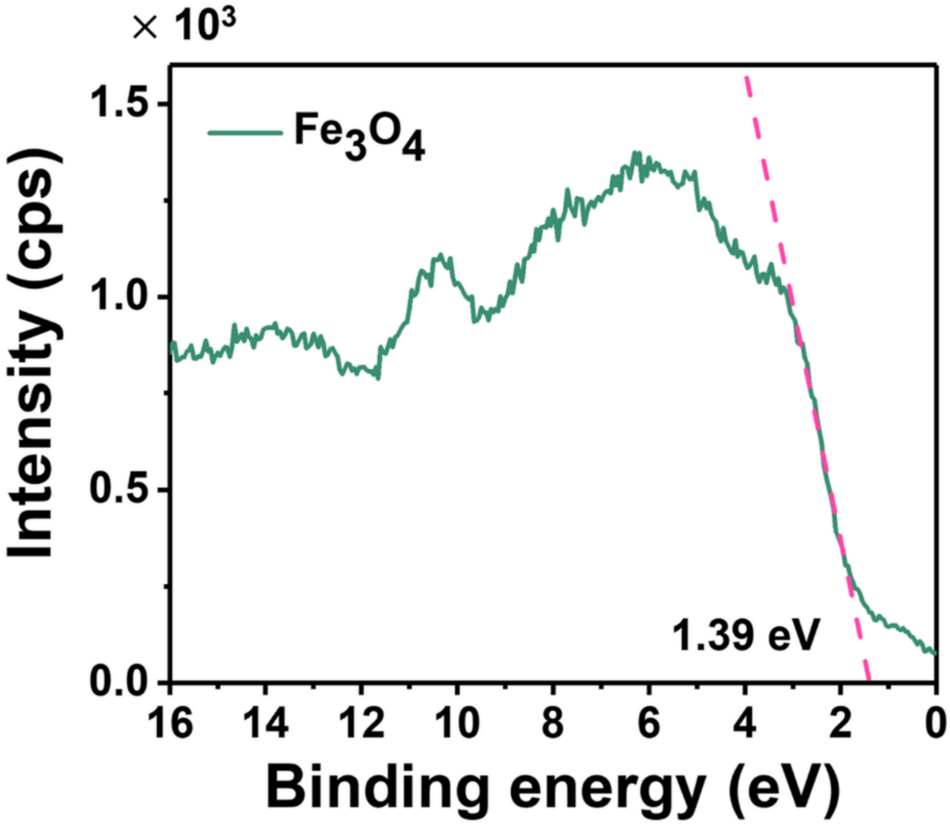

Figure S11. X-ray photoelectron spectroscopy (XPS) showed the valence band energy of Fe_3O_4 nanoparticles in the initial region.

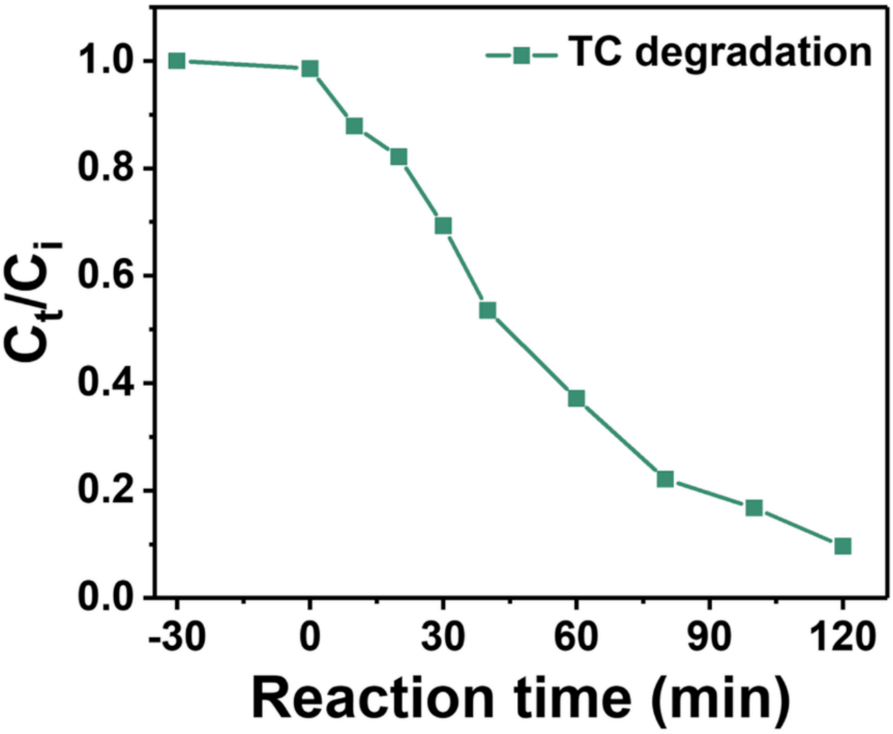

Figure S12. The photo-Fenton degradation of tetracycline employing the PFAB nanocomposites (Tetracycline = 10 mg L^{-1}, catalyst dose = 0.05 g L^{-1}, H_2O_2 concentration = 1 mм, initial pH = 3.0).
